# Microalgal Cell Biofactory—Therapeutic, Nutraceutical and Functional Food Applications

**DOI:** 10.3390/plants10050836

**Published:** 2021-04-21

**Authors:** Boda Ravi Kiran, S. Venkata Mohan

**Affiliations:** Bioengineering and Environmental Sciences Lab, Department of Energy and Environmental Engineering (DEE), CSIR-Indian Institute of Chemical Technology (CSIR-IICT), Hyderabad 500 007, India; ravikiranboda89@gmail.com

**Keywords:** bioactive compounds, food supplements, health and nutrition, immunostimulants, algal metabolite extraction

## Abstract

Microalgae are multifaceted photosynthetic microorganisms with emerging business potential. They are present ubiquitously in terrestrial and aquatic environments with rich species diversity and are capable of producing significant biomass. Traditionally, microalgal biomass is being used as food and feed in many countries around the globe. The production of microalgal-based bioactive compounds at an industrial scale through biotechnological interventions is gaining interest more recently. The present review provides a detailed overview of the key algal metabolites, which plays a crucial role in nutraceutical, functional foods, and animal/aquaculture feed industries. Bioactive compounds of microalgae known to exhibit antioxidant, antimicrobial, antitumor, and immunomodulatory effects were comprehensively reviewed. The potential microalgal species and biological extracts against human pathogens were also discussed. Further, current technologies involved in upstream and downstream bioprocessing including cultivation, harvesting, and cell disruption were documented. Establishing microalgae as an alternative supplement would complement the sustainable and environmental requirements in the framework of human health and well-being.

## 1. Introduction

Prognoses speculate the world population to reach almost 9.5 billion by 2050 and the food produced must be increased two-fold to meet the global necessities [1]. Escalating agricultural area, crop rotation, and yield increase could meet the rising demands for food; nevertheless, these practices deepen the existing environmental problems i.e., deforestation, soil degradation, and loss of biodiversity [2,3,4]. In this perspective, microalgae are emerging as a sustainable feedstock and alternative food source [5]. The primordial theory suggests that life began in a water body (ocean/ponds) and within the earth’s biome, algal strains are characterized by high biodiversity [6]. Microalgae present in freshwater and marine environments are unicellular, microscopic, and photosynthetic microorganisms. Rapid proliferation rate, high photosynthetic activity, CO_2_ sequestration, biomass production, and ability to grow on wastewater makes algae an amenable microbe for the production of value-added biochemicals [7,8,9]. Globally, 7000 tons of dry algal biomass is produced with a market value of 3800 to 5500 million USD per annum [10]. Utilizing microalgal biomass for developing pharmaceuticals, nutraceuticals, and functional foods for health benefits is rapidly soaring. For example, *Chlorella* and *Arthospira* species (2000 and 5000 tons/year) are dominant dietary supplements with a global production of USD 40 million/year [11]. The worldwide *Spirulina* market is valued at USD 629.6 million, and it is anticipated to reach a CAGR of 9.4% by 2025 [12]. Bioactive compounds synthesis has spread all over the world including countries with higher temperatures as well as longer-sunlight-duration countries (Southern Asia, Middle East, Central, and South America), which allows for more cost-effective cultivation of microalgae [13]. This communication will make an attempt to unravel the significance of microalgae metabolites in relation to dietary supplements, therapeutic activity, and utilization as feed in poultry and aquaculture farming. The cultivation, downstream process, pre-treatment, and product fractionation were also reviewed.

## 2. Potential of Microalgae

The global nutraceutical and pharmaceutical markets in 2017 were worth USD 200.2 billion and USD 934.8 billion, respectively, and are expected to hit USD 317.3 billion and USD 1170 billion by 2024, respectively [14,15]. Microalgae are essential life forms, which produce ~50% of atmospheric oxygen and function as a backbone of the food web along with bacteria supplying energy to all trophic stages [16]. Utilizing wastewater as nutrients and processing value-added products on non-arable land helps to avoid competition with food crops [17]. From a compositional viewpoint, microalgae are rich in carbohydrates (50% of dry biomass), and the absence of lignin makes them highly amenable to access sugar content without any pretreatment, which is a prerequisite in plant materials [6]. Since World War II, they have been well known for their possible bioactive compounds that support humankind [18]. Bioactive compounds occur as a part of the food chain with functional properties.

According to an estimate, there are around 200,000 to 800,000 species of microalgae and more than 15,000 novel algal biomass compounds that have been identified [19]. Microalgae are light-driven cell factories that synthesize bioactive compounds from primary metabolites (lipids, proteins, and carbohydrates) and secondary metabolites (pigments, carotenoids, vitamins, and sterols) at various growth stages. *Spirulina*, *Porphyridium*, and *Scenedesmus* are excellent sources of protein (60–70% *w*/*w*), carbohydrates (40–60% *w*/*w*), and lipids (40% *w*/*w*) [5]. These metabolites are produced from microalgae via mevalonate/non-mevalonic, shikimate, and polyketide pathways. Proteins, lipids, and carbohydrates synthesized in the growth phase act as energy reservoirs during nutrient deficient conditions [20,21]. Each microalgal species has diverse characteristics and produces various products influenced by both biotic and abiotic stress.

Microalgae produce various therapeutically active bio-compounds either from biomass/de-oiled mass or unleashed directly into the extracellular matrix [22]. Phenylalanine ammonia-lyase, an enzyme active in the biosynthesis of polyphenol compounds (phenylpropanoids, flavonoids, and lignin) was characterized by X-ray crystallography in *Anabaena variabilis* and *Nostoc punctiforme* [23]. Reactive oxygen species (ROS) that inflict oxidative damage to proteins, lipids, and nucleic acids contribute to cancer, atherosclerosis, rheumatoid arthritis, heart disease, Alzheimer’s, Parkinson’s, and accelerates aging in humans. Microalgal metabolites have potent biological processes viz. antioxidant, anti-inflammatory, antifungal, anti-microbial, anti-enzymatic, antiviral, anticancer, anti-coagulant, and immunosuppressant effects, which help in the reduction and prevention of diseases [24]. Microalgae are renowned as a rich source of biological metabolites with applications in pharmaceuticals, food, feed, and skincare products. Furthermore, the elemental microalgae abundance in terms of C, H, O, and N is two-times higher than wood and acts as a notable organic fertilizer [25]. Primal Healthcare, Rincon pharmaceuticals, Agri Life SOM Phytopharma, and Novo Nordisk India Pvt. Ltd., use micro- and macroalgae bioactive compounds in vaccine development that confer immunity against infectious diseases [11].

## 3. Bioactive Compounds

Scientists are diligently exploring to increase algal biomass especially rich in nutraceutical substances that can be utilized as whole cell or extracts and added as ingredients in foods and beverages (Table 1).

### 3.1. Chlorophyll

Chlorophyll is an omnipresent pigment (lipid-soluble) crucial for photosynthesis and in general found in all plants, algae, and cyanobacteria. Green microalgae *Chlorella* sp. is popularly called “Emerald food” because of its high chlorophyll content [26]. Chlorophyll *a*, *b*, or a standalone mixture exhibited chemo-preventive effects such as elevated glutathione S-transferase levels, cytochrome P450 enzyme inhibition, and cellular differentiation; albeit mitotic arrest and necrobiosis [27]. Chlorophyll gained significance as a dyeing agent in food and due to chemotherapeutic potential because of its derivatives in the field of medicine [28]. It is widely used as a constituent in health and hygiene products such as antiperspirants, air fresheners, lozenges, and in formulations against foul smells.

### 3.2. Carotenoids

Carotenoids are hydrophobic, light-harvesting accessory pigments with the C_40_ backbone structure of isoprene units, which act as antioxidants and play a major role in quenching free radicals and inhibiting oxidative injury to cells, tissues, and membranes [29]. Approximately 400 carotenoids were identified in different living beings, and those that are widely commercialized are β-carotene, astaxanthin, lutein, fucoxanthin, zeaxanthin, antheraxantin, violaxanthin, neoxanthin, loroxanthin, diadinoxanthin, diatoxanthin, and siphonein [30]. The global market value of carotenoids was USD 1.5 billion in 2017 and should reach USD 2.0 billion by 2022 at a compound annual growth rate of 5.7% (www.bccresearch.com, accessed on 15 March 2021). *Botryococcusbraunii*, *Chlamydocapsa* sp., *Chlamydomonas nivalis*, *Chlorella sorokiniana*, *Chlorococcum *sp., *Dunaliella salina*, *Dunaliella tertiolecta*, *and Paeonia obovate*, belonging to Chlorophyceae and Trebouxiophyceaeare are dominant microalgal species-rich in β-carotene [31]. Astaxanthin (ASX) is a red xanthophyll pigment and is the most industrially exploited ketocarotenoid with a market value of USD 200 million [32]. Astaxanthin exhibits greater antioxidant function, i.e., 100 times higher than alpha-tocopherol, 6000 times as much as vitamin C, 800 times as much as coenzyme Q10, 550 times as much as vitamin E, 200 times as much as polyphenols, 150 times as much as anthocyanins, and 75 times as much as alpha-lipoic acid [33]. Lutei is a golden-colored carotenoid with an annual production of 70–150 tons/ha, i.e., 11.5–25 times that of marigold (6 tons) and a market value of USD 3.14 million [34]. It exhibits higher antioxidant activity than β-carotene in attenuating oxidative damage and preventing damage to lipid bilayers [35]. The global market of fucoxanthin was USD 600 million in 2020 and is anticipated to reach USD 780 million by the end of 2025, growing at a compound annual growth rate of 6% between 2020 and 2025 [36]. It was first commercially produced by Algatechnologies Ltd. in 2018 with a trademark Fucovital^®^mainly employed in bodyweight management products.

### 3.3. Lipids

Lipids are essential macromolecules and critical constituents of energy reservoirs and signaling, molecules crucial for the integrity and functionality of cellular membranes. Algae contain most of the plant lipids such as glycosylglycerides and phosphoglycerides [37]. Microalgae lipid content varies from 1 to 40%, and it may rise to 85% of total dry weight depending upon nutrient composition, temperature, light flux, and CO_2_ concentration [38]. Storage lipids (triacylglycerols) are produced via photosynthesis and stored in cells, whereas fats involved in cell structure and metabolism are known as structural lipids (monounsaturated fatty acids (MUFA) and poly monounsaturated fatty acids (PUFA)). PUFAs are long unsaturated hydrocarbons with more than one double bond and have potent applications in feed and nutraceutical industries. They play a prominent role in balancing membrane fluidity, electron and oxygen transport, thermal adaptation, as well as cellular and tissue metabolism [39]. Microalgae are rich in long-chain PUFAs such as γ-linoleic acid (18:3), arachidonic acid (20:4), eicosapentaenoic acid (20:5), and docosahexaenoic acid (22:6). Docosahexaenoic acid (DHA) and eicosapentaenoic acid (EPA) are abundant omega-3 fatty acids with a market value of 80 to 160 USD kg^−1^ and the global value is predicted to be USD 898.7 million by 2025 [22]. Humans are unable to synthesize lipids themselves endogenously, and external administration in diet is crucial for maintaining hemostasis [40]. PUFA reduces the prevalence of several chronic diseases and has proven to have several health benefits, i.e., the central nervous system and human vision reinforced by FDA (Food and Drug Administration) and FAO (Food and Agricultural Organization) [11]. The balanced diet ratio of polyunsaturated fatty acids to saturated fatty acids recommended by the World Health Organization (WHO) and the FDA should be above 0.4 and prescribed intake should be 0.2–0.3 g DHA and EPA per day for a healthy individual [41]. Dihomo-γ-linolenic acid (DGLA), a trace amount long chain PUFA is important in developing infant formulations [36]. *Chaetoceros calcitrans*, *Crythecodinium cohnii*, *Isochrysis galbana*, *Monodus subterraneus*, *Nannochloropsis* sp., *Pavlova salina*, *Phaeodactylum tricornutum*, *and Porphyridium cruentum* are some of the PUFAs producing microalgal species [42,43].

### 3.4. Carbohydrates

Carbohydrates are major intracellular components acquired from photosynthesis and can reach up to 50% of microalgae dry weight. Carbohydrates are synthesized in the form of reducing sugars like glucose, lactose, sucrose, fructose, and polysaccharides [44]. Polysaccharides have attracted widespread attention from bioengineering and pharmaceutical companies as they are natural, biodegradable, non-toxic, and biocompatible. These provide health benefits by boosting the immune system, blocking tumorigenesis, and were first exploited for their rheological property as thickening or gelling agents [11]. *β*-1,3 glucan, an agile immunostimulator from *Chlorella*, alleviates hydroxyl radicals, atherosclerosis, gastric ulcers, cholesterol, constipation, and hypercholesterolemia [45]. Polysaccharides, namely immulina and immurella isolated from *Spirulina platensis* and *Chlorella pyrenoidosa*, exhibited high anticancer activity when compared to fungal polysaccharides such as schizoplyllan, lentinan, and krestin [46]. Animals fed with polysaccharide-rich algae exhibited lower cholesterol levels in serum with the subsequent reduction in insulin and glucose [47]. Sulfate ester-containing algal polysaccharides are classified as sulfated polysaccharides and are mainly produced by *Chlorella vulgaris* and *Scenedesmus quadricauda*. These polysaccharides consist of 3-linked β-D-galactose and 4-linked ά-D-galactose as alternate remnants in their backbone [48]. Sulfated polysaccharides produced from *Porphyridium* aerugineum act as a coating material on the surface of sanitary items to prevent infections from epidemic diseases like COVID-19 [49].

### 3.5. Proteins

Proteins are building blocks and essential macro-nutrients responsible for the overall growth of human body. Compared to animal-based proteins (42–52 m^2^ for chicken, 47–64 m^2^ for pork, and 144–258 m^2^ for beef production), microalgae proteins (<2.5 m^2^ per kg of protein) require minimal land for cultivation [50]. Algal proteins are used as a dietary supplement (tablets, powder, and paste), and the amino acid composition is equivalent to high-quality protein foods such as β-lactoglobulin, tofu, mushrooms, soybean, and egg white [51]. *Chlorella* sp., *Dunaliella* sp., and *Arthrospira* sp. produce top-notch proteins and amino acid profiles according to FAO/WHO guidelines and are legally marketed as nutraceuticals or fortified foods to prevent health disorders and abnormalities in cells. *Spirulina* contains 50–70% protein (dry weight) and essential amino acids, such as isoleucine, leucine, and valine, which exhibit high digestibility (83–90%). A spoonful of *Spirulina* (7 g), of which dried biomass contains almost 4 g of protein, is also known as a “superfood” by the World Health Organization [17]. Hainan Simai Pharmaceutical Co. Ltd., Haikou in China annually produces around 3 × 10^3^ tons of *Spirulina* biomass. Cyanotech (Hawaii, USA) under the name Spirulina Pacifica produces and markets products (http://www.cyanotech.com, accessed on 2 September 2009). Microalgas Macronutricao factory in Brazil produces *Spirulina* lozenges for dietary nourishments (http://www.olson.com.br, accessed on 14 July 2001). Phycobiliprotein are hydrophilic protein–pigment complexes present only in cyanobacteria. These are used as fluorescent labels in antibodies and receptors, flow-cytometry, immunohistochemistry, immunoblotting experiments, and microscopy or fluorescence diagnosis [52]. Triton Health and Nutrition (USA) and Algenics (France) are the first manufacturers to use microalgae in high-throughput recombinant protein expressions. *Chlamydomonas reinhardtii* and *Chlamydomonas elipsoidea* contain recombinant proteins viz. vascular endothelial growth factor (VEGF), erythropoietin, Human IgG αPA83, hGAD65, immunotoxin αCD22-PE40, Plasmodium surface proteins (AMS1, MSP1, VP28, Pfs25, Pfs28, CtxB-Pfs25 and Pfs 48/45), Anti-CD-22-gelonin, lsc αHSV glycoprotein D, CSFV-E2, hTRAIL, and Anti-PA 83 anthrax IgG1, which possess therapeutical applications in anemia treatment, wound healing, anti-malarial vaccines, and as an antibody against anthrax, Herpes simplex virus, human papillomavirus, and foot-and-mouth disease [10,53,54]. Besides these, it functions as an immunotoxin against B-cell lymphoma and has increased resistance to UV-induced stress [55].

### 3.6. Vitamins

Microalgae are the cradle homes of vitamins that can be used as ingredients or food supplements to nourish the human body, revitalize cells, detoxify, as well as activate the immune system [56]. Vitamin A (β-carotene), vitamin C, E, and B i.e., thiamine (B1), riboflavin (B2), niacin (B3), pantothenic acid (B5), pyridoxine (B6), folic acid (B9), and cyanocobalamin (B12) are commonly produced vitamins. Vitamin B12 and provitamin A (β-carotene) are rich in *Spirulina*, which upon consuming, improves *Lactobacillus* in the intestine and makes it possible to digest vitamin B1 and other vitamins from food more efficiently [57]. Lutein and Zeaxanthin synthesized from *Spirulina maxima*are rich in vitamin A and act as protective factors from harmful light sources and age-related macular degeneration (AMD) [41]. *Anabaena cylindrica*, a cyanobacterium, produces a high concentration of vitamin K1 (200 µg g^−1^), which plays a significant role in the inhibition of chronic ailments [58]. Microalgae containing vitamin D_2_ and D_3_ together with provitamin D_3_ acts as a chemopreventive agent and exerts antiproliferative and immunomodulatory activity on tumor-growing cells [59]. Vitamin E synthesized in microalgal species such as *Chaetoceros calcitrans*, *Dunaliella tertiolecta*, *Nannochloropsis* sp., *Porphyridium *sp., and *Tetraselmis suecica* improves endothelial function, vascular health, and inhibits prostate cancer cell growth [60].

### 3.7. Sterols

Phytosterols are structurally similar and functionally analogous to cholesterol with an additional alkyl group in the side chain of the sterol nucleus. They are used as additives in many food products and characterized by the presence of different phytosterols such as stigmasterol, sitosterol, and brassicasterol [20]. Sterols are essential constituents of cell membranes and play a key role in stabilizing phospholipid bilayers and in signal transduction as hormone precursors. These are crucial components of a healthy diet for reducing blood cholesterol levels in hyper and normocholesterolemic people and inhibiting colon cancer development [61]. Methanolic extracts of *Chlorella vulgaris* exhibited anticancer and anti-inflammation properties due to the presence of 2 delta (5, 7)-sterols (ergosterol and 7-dehydroporiferasterol peroxide), 7-oxo-delta(5)-sterol (7-oxocholesterol), and 5,8 alpha-epidioxy-delta(6)-sterol [62]. *Pavlova lutheria*, *Nanochloropsis* sp. (BR2), and *Tetraselmis* sp. (M8) are good phytosterol producers, especially *Pavlova lutheria*, with 5186 mg/100 g. The minimal intake of 2 to 3 g day^−1^ promotes the smooth functioning of the heart, blood vessels, and minimizes lipoprotein [63].

## 4. Therapeutical Function of Microalgae

Microalgae and their extracts are an enormous and under-explored source of biologically active compound (Table 2).

### 4.1. Antioxidant Activity

Prooxidants are endobiotic or xenobiotic that induce oxidative stress either by generation of reactive oxygen species (ROS) or by inhibiting antioxidant systems situated in the inner mitochondrial membrane. Reactive oxygen species (ROS) produce intermediates such as superoxide anion (O_2_^−^), singlet oxygen (^1^O_2_^−^), hydrogen peroxide (H_2_O_2_), peroxyl (ROO^.^), and hydroxyl radical (-OH), which are accumulated due to the imbalance in the equilibrium ratio of oxidant to antioxidant [12,87]. They are intruded upon as a result of smoking, unhealthy eating habits, exposure to sunlight, UV irradiation, X-rays, and gamma rays. ROS/Reactive nitrogen species (RNS) may lead to increased oxidative stress and are responsible for membrane lipid peroxidation, swelling and lysis of mitochondria, mutagenic actions, and post-translational protein modifications including various chronic diseases like diabetes mellitus, Alzheimer’s, respiratory disorders, rheumatoid arthritis, cataracts, Parkinson’s, cancer, as well as the aging process [88]. The antioxidants synthesized within the human body in vivo and incorporated by enzymatic and non-enzymatic pathways are called endogenous antioxidants. Antioxidants supplemented through ex-situ or external food supplements are called exogenous antioxidants/nutrient oxidants. They include bioflavonoids, carotenoids, vitamins (C and E), biometals, and omega-3 and omega-6 fatty acids [73]. Increasing concern over oxidative stress-mediated diseases led to the identification of therapeutic approaches and herbal medicines derived from foods rich in natural antioxidants. Microalgae are well known for natural antioxidants because of their fast growth rate and production of multiple components in a single species. Algae contain several enzymatic antioxidants (superoxide dismutase (SOD), catalase (CAT), glutathione peroxidase (GPX), and peroxiredoxin (PrxR)) and non-enzymatic antioxidants (phenols, flavonoids, and alkaloids) that quench free radicals or rejuvenate antioxidants with the aid of reducing equivalents generated by photosynthetic processes [89]. The presence of conjugated double bonds or the presence of epoxy, acetyl, allene, or acetylene groups with carotenoids are responsible for antioxidant activity [56]. Antioxidants synthesized from microalgae (Butylated hydroxyanisole (BHA) and Butylated hydroxytoluene (BHT)) provide better protection when compared to synthetic antioxidants. Seven microalgal species viz *Spirulina platensis*, *Nostoc* sp., *Anabaena oryzae*, *Anabaena flos aquae*, *Oscillatoria* sp., *Phormedium fragile*, and *Chlorella vulgaris* were screened for antioxidant activity by 2,2-Diphenyl-1-(2,4,6-trinitrophenyl)hydrazyl (DPPH) and 2′-azino-bis(3-ethylbenzothiazoline-6-sulfonate)(ABTS) methods [90]. The antioxidant property of nine microalgae varied from 30.1 to 72.4% and 31.2 to 75.9% when compared with standard BHT antioxidant (80.2% and 85.6%) [90]. Astaxanthin suppresses oxidative injury by activating quinone oxidoreductase (NQO-1), glutathione-S-transferase-alpha1 (GST-α1), and heme oxygenase (HO-1) via the antioxidant responsive element (Nrf2-ARE) and nuclear factor erythroid-related factor 2 signaling pathway [68,91]. *Chlorella sorokiniana* contains β-carotene (600 µg g^−1^), ά tocopherol (112 µg g^−1^) and lutein (4300 µg g^−1^), which possess high radical scavenging activity [92]. Lutein and *β*-carotene extracted from *Chlorella* sp. significantly inhibited cognitive disability that leads the way to Alzheimer’s disease in rats [46]. A cocktail, i.e., 100 µg mL^−1^ of *Tetraselmis suecica* hydroalcoholic extract (antheraxanthin, neoxanthin, carotenes, lutein, and violaxanthin) showed resistance to 30 mM of H_2_O_2_ in human cells [71]. Phycobiliproteins extracted from *Phormidium autumnale* exhibited an antioxidant capacity of 274 µmol Trolox g^−1^ of total dry biomass weight [93]. Sulfated polysaccharide extracted from *R. reticulata* is twice as potent as ά-tocopherol against superoxide anion [74,94].

### 4.2. Antimicrobial Activity

Microalgae are a valuable source of antibiotics and show potent antimicrobial activity because of their ability to produce carbohydrates, proteins, lipids, halogenated hydrocarbons, phytosterols, monocyclic aromatic compounds, phenols, and polyketide products [46]. Lipid structure and other compounds such as ά-and β-ionone, β-cyclocitral, neophytadiene, and phytol are attributed to the antimicrobial action of microalgae extracts. Microalgal derived composites are used as adjuvants in fortified foods and fodder formulations to reinstate existing synthetic compounds of microbial origin.

#### 4.2.1. Antibacterial Activity

The growing resistance of pathogenic bacteria for antibiotics with consequences on human health is of great concern. These bacteria contaminate foods and, in turn, infect humans causing several foodborne diseases like diarrhea [95]. Pratt was the pioneer who isolated chlorellin, a fatty acid mixture from *Chlorella*, which exhibited antibacterial activity against several Gram positive and Gram negative bacteria [96]. ‘Parsiguine,’ an antimicrobial compound isolated from *Fischerella ambigua*, showed a minimum inhibitory concentration (MIC) of 40 mg/mL and 20 mg/mL against *S. epidermidis* PTCC 1114 and *C. krusei* ATCC 44507 [97]. The extracts from algae paved a path to develop new drugs against bacterial infections, foodborne, and human pathogens. Microalgae antibacterial activity functions against many human pathogens such as *Salmonella*, *Escherichia coli*, *Campylobacter*, *Pseudomonas*, *Shigella*, and *Staphylococcus*, which can be attributed to the presence of monounsaturated and polyunsaturated fatty acids. These fatty acids possessing antibacterial activity can also impede bacterial fatty acid biosynthesis [98]. Algal fatty acids interfere with bacterial growth and cause harmful effects to the microorganisms, i.e., cell leakage, membrane damage, reduced nutrient uptake, and inhibit cellular respiration. Fatty acids with more than ten carbon atom induce bacterial protoplast lysis [99]. Gram (−) bacteria are more resistant than Gram (+) bacteria due to their complex membrane permeability and impenetrable cell wall. Methanol, benzene, petroleum ether, and hexane are the solvents used to extract antibacterial compounds from microalgae [100].

Microalgal species from different taxonomic groups contain mixed antibacterial fractions such as chlorellin, alpha-linolenic acid, polyunsaturated fatty acids, methanolic extracts, short-chain fatty acids, phycobiliproteins, exopolysaccharides, beta-ionone, and neophytadiene [101]. These fractions possess antibacterial property against numerous human bacteria, namely *Eischerichia coli*, *Pseudomonasaeruginosa*, *Enterobacter aerogenes*, *Salmonella typhimurium*, *Klebsiella pneumonia*, *Vibriocholerae*, and *Proteus vulgaris* [97,102]. Chlorophyceae and Bacillariophyceae are predominant and possess high antimicrobial activity compared to other classes of algae. *Dunaliella* sp., a green microalga isolated from wastewater streams, showed a higher bactericidal effect than its ecads. EPA, hexadecatrienoic acid (HTA) and palmitoleic acid (PA) from *Chlorococcum* strain HS-101, *Dunaliella primolecta*, and *Phaeodactylum tricornutum* (diatom) showed potent bacteriostatic activity against methicillin-resistant *Staphylococcus aureus* (MRSA), which is highly resistant to conventional antibiotics and is an increased concern in healthcare institutions worldwide [103]. Decadienal, microalgae-derived oxylipins exhibit strong antibacterial activity against *Pfeiffer’sbacillus* (IC_50_–1.9 µg/mL) and methicillin-resistant *Staphylococcus aureus* (IC_50_–7.8 µg/mL). Further, it impairs the production of different bacteria such as *Eischerichia coli*, *Pseudomonas aeruginosa*, *Aeromonas hydrophila*, *Staphylococcus epidermidis*, *Photobacterium phosphoreum*, *Planococcus citreus*, and *Micrococcus luteus* [89,98]. Short-chain fatty acids from *H. pluvialis* showed antibacterial activity against *E. coli*, whereas long-chain fatty acids from *S. obliquus* showed the same against *Staphylococcus aureus* [104]. *S. grantiana* and *O. sancta* ethanolic and methanolic extracts exhibited antibacterial activity against human pathogens, i.e., *E. coli*, *P. vulgaris*, and *P. mirabilis* with an inhibition zone of 9, 10, and 9 mm, respectively [105]. Methanol extracts of *Dunaliella salina* and *Pseudokirchneriella subcapitata* with MICs ranging from 1.4 × 10^9^ to 2.2 × 10^10^ cells/mL showed antimicrobial activity against external otitis (inflammation around the auditory canal and auricle) and indicate the evolution of *S. aureus*, *P. aeruginosa*, *E. coli*, and *Klebsiella* sp. [106]. Polysaccharides can act as surfactants and also modify bacterial morphology [107]. Microalgal polysaccharides act similar to *E. coli* polysaccharides and can inhibit bacterial adhesion and aggregation properties.

#### 4.2.2. Antiviral Activity

Viruses are the smallest infectious agents for leading death rates globally. Preventing the invasion or synthesis of viral components is ambiguous as they have a deleterious impact on the host system. Although several antiviral drugs originated from plant secondary metabolites, mammals, and synthetic chemicals, drug-resistant mutations are continually occurring. Microalgae have gained renowned recognition as potential sources of antiviral agents in conjunction with the promotion of blue technology [108]. In antiviral compounds, microalgae predominate and cyanobacteria are a potential source of antiviral agents on an industrial scale [109]. Polysaccharides isolated from *G. cartilagenium* were the first antiviral compounds to safeguard egg embryos from mumps and flu viruses [110]. Herpes simplex virus (HSV1/HSV2) is a prevailing contagious disease, and around 30% of the global population is affected by genital and oral herpes. Several studies documented that marine polysaccharides present a wide antiviral spectrum against enveloped viruses. Spirulan, a sulfated polysaccharide isolated from *Spirulina platensis*, demonstrated good antiviral activity against human immunodeficiency virus (HIV1) and herpes simplex virus (HSV1) [111].

Acyclovir^®^, an antiviral compound isolated from *Dunaliella sp*., also deactivates the viral infections of HSV and HIV-1. Higher sulfate content induces higher antiviral activity, and the inhibitory effect arises by blocking the receptor sites of viral glycoprotein [74]. Polysaccharides (PS) isolated from *A. platensis*, *R. reticulate*, *Porphyridium *sp., *G. impudicum*, and *C. polykrikoides* are the prominent microalga exhibiting antiviral properties against innumerable viruses like *Human alphaherpesvirus 3* (HHV-3), *Herpes simplex virus* (HSV1/HSV2), Human beta herpesvirus, murine leukemia virus (MuLV), Flu-A viruses, Hepatitis B virus (HBV), Encephalomyocarditis virus, Flu-A and B, viral hemorrhagic septicemia virus (VHSV), measles, African swine fever virus (ASFV), vaccinia virus (VACV), mumps, vesicular stomatitis virus (VSV), and respiratory syncytial virus (RSV-A& B) [101,112,113,114,115]. Cyanovirin, a potential protein moiety produced by *Nostoc* species, showed promising effects in curing human influenza A and HIV [82]. The Algevir technology expressed an antigenic protein called ZK from *Schizochytrium* sp. in the Zika virus (ZIKV) glycoprotein envelope and displayed substantial humoral responses to those caused by subcutaneous immunization at greater magnitudes upon oral administration [81]. Extracted from *Gyrodinium impudicum*, sulfated polysaccharide p-KG03 demonstrated strong efficacy against the encephalomyocarditis RNA virus (EMCV) relative to current medicines [80]. Polysaccharide (TK V3) and exopolysaccharide from *A. platensis* and *P. purpureum* were shown to be successful against Vaccinia and Ectromelia orthopoxvirus with IC_50_ values (0.78 and 0.65 mg/mL) when examined with HEp-2 and Vero C1008 cells lines [116]. Naviculan isolated from diatom *N. directa* and A1/A2 from *C. polykrikoides* showed strong antiviral activity against contagious diseases like HIV1, HSV1, and type A flu viruses [117]. Carrageenan lozenges are used for treating patients with sore throat and carrageenan pills are particularly potent against HRV1a HRV8, influenzavirus A H1N1n, Coxsackievirus A10, and human coronavirus (hCoV) OC43 [72]. Similar studies were carried out by Graf et al. for treating rhinitis and sinusitis caused by human rhinovirus 1a and human coronavirus OC43 [118]. The viral hemorrhagic septicemia virus (VHSV) affects more than 50 species of commercially important freshwater and marine fish, including salmonid fish. Nevertheless, fewer studies were conducted on the antiviral properties of microalgae.

#### 4.2.3. Antifungal Activity

Fungal contagions were mostly due to intensive chemotherapy regimens that depress the patient’s immune system, organ transplantation, and spread of prevalent diseases [119]. Screening of microalgae for antifungal compounds began far beyond antibacterial activity. Aspergillosis induced by *Aspergillus sp*. and candidemia caused by *Candida sp*. has been increasing exuberantly since ancient periods [120,121]. The formulation of new drugs and antifungal activity varies widely depending on microalgal species. Marine microalgae (e.g., *Chaetoceros* sp.) have more capability in developing novel antifungal compounds compared to fresh habitats [102]. Several antifungal compounds such as polysaccharides, organic solvent extracts, polyethers, and lipid fractions isolated from green (*H. pluvialis*, *Chlorella vulgaris* and *Scenedesmus* sp.) and red microalgae (*Porphyridium* sp. and *R. reticulata*), Bacillariophytes, and dinoflagellates show activity against microorganisms such as *A. fumigatus*, *A. niger*, *Penicillium* sp., *Saccharomyces*sp., *Candida* sp., *Microsporum* sp., and *E. floccosum* [122,123]. Gambieric acid, a potent antifungal agent with polycyclic ether isolated from *Gambierdiscus toxicus*, acts against molds and dermatophytes while the diatom *Thalassiothrix frauenfeldii* was involved against active yeasts [124]. Amphidinol, a potent antifungal agent isolated from *Amphidinium klebsii*, targets fungi membrane, mainly the ergosterol biosynthetic pathway [125]. Pigments produced by *H. karadagensis* showed antifungal activity against *C. maritima*, *D. salina*, and *Lulworthia* sp. responsible for biological fouling [126]. Organic extracts from *C. lauderi*, *G. toxicus*, and *C. vulgaris* inhibit *Aspergillus fumigatus*, a toxin-producing fungus that contaminates seafood of marine bivalves [127]. 

### 4.3. Anticancer Activity

Cancer is the single most serious disorder causing irregular cell growth that has the ability to infiltrate other body parts. A lump, irregular bleeding, excessive coughing, weight loss, and a decrease in bowel movements are potential symptoms. Human beings are affected by over 100 types of diseases, the bulk of which are due to hereditary abnormalities, i.e., environmental and lifestyle causes (90–95%), and the remaining 5–10% is due to inherited genetics [128]. Chemotherapy is the most promising anticancer approach aimed at reducing mortality by delaying the carcinogenesis process. Several enzymatic and non-enzymatic antioxidants from natural sources suppress tumorgenesis by inhibiting tumor cell protease activity, fostering cell division, triggering cell death, and immunomodulations [83]. Chinery et al. reported that the combined effect of antineoplastic agents and antioxidants helps in remission of colon cancer [129]. Cyanobacteria are an abundant source of natural foods, and their medicinal value was recognized at the beginning of the 15th century when *Nostoc* strains were used to treat malignant tumors, arthritis, and fistula. *Anabaena* and *Nostoc* sp. are important cyanobacteria that produce over 120 natural products with potent activity against several cancer-causing agents [130]. Chlorophyllin, a derivative of chlorophyll, acts against HCT1 16 human colon cancer cells because of its high effectiveness as a chemo-preventive agent [64]. Aqueous extract of *Nostoc muscorum* showed the highest anticancer activity in humans against Ehrlich cell (87.25%) and hepatocellular carcinoma (89.4%) [90]. This is attributed to the high content of phycobiliproteins, polysaccharides, and polyphenols, which induced programmed cell death of tumor cells. Bioactive compounds dragonamide A-E and dragomabin isolated from cyanobacterium *Lyngbya *sp. exhibited in vivo activity against leishmaniasis. Moore’s group isolated twenty-six cryptophycin from *Nostoc *sp. GSV 224, of which cryptophycin 52 was reported to be successful in lab trials for treating ovarian carcinoma and alveoli cancers [131]. C-phycocyanin, an aqueous extract of red algae, exhibits antiproliferation inducing apoptosis [132]. β-(1, 3)-glucan, an active immunostimulator from *C. vulgaris*, is used as a potential antitumor agent [133]. Sulfated polysaccharides isolated from *P. cruentum* exhibited intense antitumor activity in rhodent peritoneum by hindering cancer cell progression and increasing the spleen and thymus indexes as well as boosting the immune system [134]. Exopolysaccharides of *Porphyridium *sp. retarded neoplasia, and its biomass in rats prevented the growth of colon cancer [135]. Fewer reports supported that gastric cancer can be treated using microalgae-derived peptides [136]. Vitamin B12, isolated from marine algae, is used as an encapsulating agent for anti-cancer drugs and plays a vital role in histone methylation, DNA synthesis, and the regeneration of blood cells. Cobalmin and folate extracted from diatomous microalgae with high proportions lower the risk of breast cancer [86].

### 4.4. Anti-Inflammatory

Inflammation is a cell or tissue’s biological reaction to harmful factors, such as pathogenic agents, pollutants, weakened cell, and irritants, as well as a defensive response containing skin cells, blood cells and molecular mediators [137]. Inflammation normally occurs in heat, pain, swelling, and loss of control at the site of infection, but diabetes, obesity, renal, cardiovascular, and neurodegenerative disorders may be caused by chronic inflammation [138]. Oxidative stress is the predominant factor responsible for several inflammatory responses inducing mitochondrial dysfunction and beta-amyloid aggregation [139]. Besides, ROS generated in brain tissue also causes neurodegeneration, cellular death, and memory loss by modulating synaptic and non-synaptic communication between neurons [140]. Oral uptake/consumption of anti-inflammatory compounds revitalizes body immunity and aids in the healing process. Microalgae produce several anti-inflammatory metabolites such as carotenoids, PUFAs, phycobiliproteins, carbohydrates, and sulfurized polysaccharides that suppress chronic inflammation and exert a protective function when consumed as food or used as pharmaceutical supplements [141,142]. Because of the metabolites mentioned above, microalgae are being considered in regenerative medicine for developing tissue scaffolds, especially in patients with skin burns [143]. The PUFAs (*ω*3 and *ω*6 fatty acids), especially arachidonic, docosahexaenoic, and eicosapentaenoic acids, are mainly involved in treating rheumatoid arthritis and dermatitis. Anti-inflammatory effects of DHA are prominent in developing breast milk, human fetus, and act against colon and breast cancer [144]. DHA and EPA produced from *Schizochytrium* species are widely used for nourishment in women in maternity and cardiac patients. DHA intake as a subsidiary food modulated fibrillar oligomers in transgenic APP/PS1 rat brains by blocking βeta-amyloid aggregation [77]. Docosapentaenoic acid (DPA) extracted from *Schizochytrium *sp. inhibits cytokines (IL-1 and TNF- α) in peripheral blood mononuclear cells (PMBC) of humans [145]. Astaxanthin produced from *Haematococcus pluvialis* had more remarkable inhibitory effects on lipopolysaccharide-induced inflammation and anti-inflammatory action than prednisolone [146]. ASX could prevent lipopolysaccharide-induced acute respiratory distress syndrome (ARDS) and sepsis by suppressing the signaling pathway of MPAK/NF-KB and decreasing pro-inflammatory cytokine level [65]. It also showed protective effects against lung injury, repressed alveolar wall swelling, myeloperoxidase activity, and attenuation of number of pulmonary alveoli in lung tissues [32]. Research conducted by Chang et al. reported that astaxanthin defends nerve cells against Aβ25-35-induced apoptosis by inhibiting radical oxidative destruction, Bax expression, nuclear transcription factor-kB (NFkB), and P-38 MAP kinase dephosphorylation (mitogen-activated protein) [66]. In a clinical trial performed on healthy young women, a daily dosage of two milligrams of astaxanthin for two months lowered inflammatory cytokines, C-reactive protein, and also decreased ROS production by the transcription factors regulating nuclear factor (NF-κB) and activator protein (AP-1) [67]. Algal metabolites produce various pro-inflammatory mediators, and its mechanism includes enzyme modulation (prostaglandin-endoperoxide synthase, phospholipase A2, and NO synthase), modulating cellular processes, and intervening with nuclear factor κB (NF-κB) and the mitogen-activated protein kinase pathways [147,148]. Inhibitory effects and reduced production of nitric oxide synthase and prostaglandins in phagocytic cells were shown by Fucoidan, a sulfated polysaccharide isolated from brown algae [149]. Among the pigments, fucoxanthin isolated from Bacillariophyceae is capable of eliciting metastasis in humans, and phycobiliproteins from cyanobacteria can inhibit histamine release [150]. Spirulan isolated from *A. platensis* showed similar properties to that of glycosaminoglycans (GAG), present in animals with bioactivities involved in human physiological and pathological responses [151]. Besides oxidative stress, exopolysaccharides from *Porphyridiumcruentum* enhanced immunostimulating activity in vitro and inhibited membrane peroxidation [134]. Sulfated polysaccharides possessing anti-inflammatory properties can be used in dermal treatments by blocking the polymorphonuclear leukocytes stimulations. Sulfolipids isolated from *Scenedesmus rubescens*, *Scenedesmus acuminatus*, and *Phaeodactylum tricornutum* are effective in inhibiting alpha-glucosidase, glutaminyl-peptide cyclotransferase, and telomerase activities [73]. In addition, natural ASX, with its proven anti-inflammatory and anti-oxidant activity backed by multiple preclinical and human trials and with its extraordinary safety profile, can be one of the most promising candidates to be tried against COVID-19 [32].

### 4.5. Antiaging

Bioactive compounds of micro- and macroalgae are compounds of interest in cosmetics and skin care products. They accelerate pigmentation, skin moisture, prevent blemishes, help seborrhea, atopic dermatitis, and skin carcinogenesis [50]. Fujimura’s group in 2002 first identified that aqueous extracts of *Fucus vesiculosus* (Bladder wrack) improved skin thickness and cheek elasticity. Algal extracts increase the transcriptional level and expression of involucrin (INV), loricrin (LOR), transglutaminase-1 (TGM-1), filaggrin (FLG), and type 1 pro-collagen, which are the major markers for skin barrier function [152]. Mycosporine amino acids (MAA) and polysaccharides are widely employed in skin photoaging, and their efficiency was tested in HeLa cervical adenocarcinoma and B16-F1 murine skin melanoma of human cell lines [84]. MAAs have high efficiency in absorbing UV radiation (310–360 nm) with molar absorptivity ranging from 2.8 to 5.0 × 10^4^ M^−1^ cm^−1^ [36]. Pentapharm (Basel, Switzerland), Blue Retinol^TM^, Protulines, Exsymol S.A.M., (Monaco, Europe), and Helioguard^®^365 and SILIDINE^®^ (Greentech, New Jersey, USA) are popular anti-aging skin products extracted from *Nannochloropsis oculata*, *Porphyridium cruentum*, and *Arthrospira* sp. [78]. Solazyme Inc. (San Francisco, CA, USA) and Terravia Holdings, Inc. (San Francisco, CA, USA) combined *Parachlorella* exopolysaccharides with *Chlorella* biomass and launched Golden Chlorella™ and AlgaPür™ algae oils that claim to deliver strong cosmetic benefits to the skin and hair [153].

### 4.6. Other Activities

Hypertension, including diabetes, kidney failure, stroke, and coronary diseases, is one of the leading risk factors for many chronic illnesses. Blood pressure in the human body is regulated by the angiotensin I conversion enzyme (ACE) found in the renin-angiotensin-aldosterone system [154]. Captopril, lisinopril, and enalapril are commercially available synthetic ACE inhibitors that are used for treating hypertension. This showed numerous side effects such as coughing, dizziness, fatigue, headaches, and angioedema [155]. Widely used microalgae to produce ACE-inhibitory peptides are *Chlorella vulgaris*, *Nannochloropsis oculata*, *C. ellipsiodea*, and *Arthrospira platensis*. Dermochlorella DG, a product derived from *Chlorella vulgaris* and *Arthrospira* proteins, activates collagen synthesis, reduces striae distensae, vascular lesions, and also acts as skin resurfacing agent [11]. A purified peptide sequence isolated from *C. vulgaris* (Val-Glu-Cys-Tyr-Gly-Pro-Asn-Arg-Pro-Gln-Phe) and *C. ellipsoidea* (Val–Glu–Gly–Tyr) showed IC_50_ values of 29.6 µM and 128.4 µM when administered orally [156]. Similarly, the peptide sequence (Gly-Met-Asn-Asn-Leu-Thr-Pro and Leu-Glu-Gln) from *N. oculata* exhibited ACE-inhibitory activity of 123 µM and 173 µM, respectively [157]. These peptide sequences bind to C- and N-terminal active sites of ACE and block Angiotensin II production and prevent organ damage. Nanofibrils extracted from *A. platensis* could be used as an extracellular matrix for treating spinal cord injuries by culturing stem cells [158]. Spirulan isolated from *A. platensis* exhibits anti-thrombogenic properties by interfering with the blood coagulation-fibrinolytic system [159]. Aeruginosins and depsipeptides isolated from *Microcystis aeruginosa* and other microalgaeshow inhibitory effects against plasmin, thrombin, trypsin, and chymotrypsin [41]. *Spriulina* extract (250 mg) plus Zinc (2 mg) twice daily helps with the detoxification of heavy metals like Arsenic [160].

## 5. Functional Food/Feed

### 5.1. Functional Food

Microalgae are considered as “GRAS” (Generally Regarded as Safe) [165] and marketed as functional foods that benefit health beyond the basic role of nutrition. *Nostoc *sp. was used in China for food (over 2000 years ago), and later *Chlorella* sp. and Spirulina sp. were consumed as healthy foods in Japan, Taiwan, and Mexico (39). Microalgae are commercially produced in the Asia-Pacific region with capacities ranging from 3 to 500 tons/year, and the market value for human consumption is 100 €/kg [166]. Currently, microalgae are available in markets as health foods, and their products are mixed with pastes, snacks, candy, gums, noodles, wine, beverages, and breakfast cereals. *Auxenochlorella protothecoides*, *Chlorella vulgaris*, *Chlorella pyrenoidesa*, *Dunaliella salina*, Dunaliella tertiolecta, and *Spirulina platensis* are some of the microalgae species widely used in human foods because of their high protein and nutritive value [167]. Besides, *Scenedesmus* sp. is also used as a nutritional food source in desserts, fruit puddings, ravioli, noodles, and soups, but their commercial production is limited [168]. In fact, the omega-3 acids in fish come from the microalgae consumed at the bottom of the food pyramid and are gradually passed up to the fish at the top [39]. *Schizochytrium* sp is cultivated by OmegaTech (USA) as an alternative to fish oil to produce DHA-rich oils, namely ‘DHA Gold’ [75]. In infant-grade milk in Europe, purified PUFAs are added, and chickens are supplemented with microalgae to produce omega-eggs [75]. In USA, EPA + DHA-rich algal oil capsules (Pure One^TM^) are marketed as food supplements [169].

### 5.2. Feed—Animal and Aquaculture

Microalgae are a treasure trove when it comes to nourishment, since they act efficiently as animal and aquaculture hatchery dietary supplements. The global microalgae production for aqua farming reached 1 kiloton (molluscs −62%; crustaceans −21%; fishes −16%) and 4.5 kilotons for ruminants and plants [170]. Moreover, microalgae is cost-effective and eco-friendly when compared to other feeds [171]. The protein content (510–710 g/kg) and essential amino acids in some microalgal species is greater than normal eggs (132 g/kg) and *Glycine max* (370 g/kg). *Spirulina platensis* exhibit a protein digestibility rate of 87–97% [172]. Microalgae-based eggs are marketed as vegan eggs by a UK company with similar nutrition to that of an egg (www.mygenefood.com, accessed on 15 March 2021). As microalgae feed is thick and recalcitrant owing to cell wall with cellulose and polysaccharides, CAZymes are used as a flavor enhancer, which helps in food assimilation [173]. Microalgae species such as *Nannochloropsis*, *Chlorella*, *Dunaliella*, *Haematococcus*, and *Schizochytrium* with sizes of 25 µM or less are used as feed for zooplankton [174,175]. Shrimps feed entirely on microalgae and zooplankton during their metamorphosis. An algal diet rich in DHA and arachidonic acid (ARA) improved hemocyte count, larvae growth, and antioxidant and bactericidal properties. It also protects against white spot syndrome virus [176]. Fish like sea bream, turbot, seabass, striped mullet, flounders, halibut, Atlantic cod, lobster, and Senegalese solely depend on microalgae as live prey/feed in hatcheries [177]. *Haematococcus pluvialis*, a rich source of astaxanthin, provides pinkening, a nutrient profile, as well as increasing the selling price of the fishes. Utilizing *H. pluvialis* as feed for farmed salmonid has been recently recommended by the Food and Drug Administration [1]. *Carassius auratus* (ornamental goldfish), when fed with microalgae, showed enhanced pigmentation. *Nannochloropsis *sp. is used in finfish hatcheries while *Pavlova* sp. is used for oyster, broodstock, clams, and mussels to enhance DPA/EPA levels [178]. *Chlamydomonas reinhardtii*, genetically engineered microalgae, produce bovine lactoferricin, increased PUFA levels, fatty acids, and anti-p57 antibodies [179]. Around 30% of total algal biomass is marketed as feed or utilized in food formulations [85].

*Chlorella* sp., *Spirulina* sp., *Arthrospira* sp., *Porphyridium* sp., and *Isochrysis* sp., are important microalgae species used in animal husbandry [173]. Canthaxanthin containing microalgae is associated with an improved content of vitamin E in the liver and is also used as a poultry dietary supplement with antioxidant, anti-inflammatory, and neuroprotective properties [41]. Holstein cows fed with microalgae produced enhanced milk yield (34 kg day^−1^), plasma metabolites, DAH, and linolenic acid [180]. *Chlorella pyrenoidosa* and *Spirulina platensis* contained essential nutrients like lactobiose, protein, and saturated fatty acids in the paunch of ruminants [181]. Sheep and Damascus goats, when fed with microalgae, showed high strength, increased protein, and lactobiose content with the same pH of the alimentary canal. Pregnant ewes supplemented with *Spirulina* gave birth to ewes 4 times larger than that of normal ones [182,183]. Poultry birds (chickens, ducks, turkeys, and quail) supplemented with microalgae reported being productive concerning body weight, yield, and reduced cholesterol [184]. Feeding chickens with *H. pluvilais*, *N. gaditana*, and *Spirulina* resulted in higher muscle pigmentation, antioxidant properties in the liver, and carotenoid pigmentation in egg yolk [185,186]. The average daily benefit and DHA levels in loin and subcutaneous meat are higher in pigs. Defatted algal biomass of *Nannochloropsis* sp., on feeding to anemic pigs, elevated blood hemoglobin, hematocrit, and growth [187]. *Drosophila melanogaster*, when fed with *Chlorella vulgaris* and *Senedesmus obliqus*, showed increased total weight, longer travel distance, and higher serotonin levels [188].

## 6. Microalgae Cultivation

**Figure 1:**
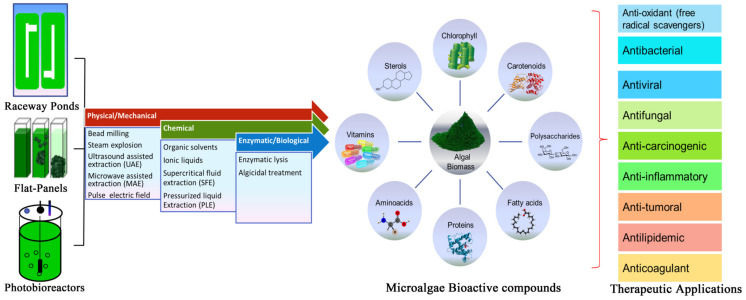
Stepwise illustration of algal biorefinery and their therapeutic activity.

Microalgae are versatile microorganisms that can grow under phototrophic, heterotrophic, and mixotrophic growth conditions and possess the ability to adapt to different environmental conditions by altering cellular mechanisms [189]. Microalgae are grown in a wide variety of indoor and outdoor cultivation systems, such as vertical column photobioreactors, raceway pools, tubular closed photobioreactors, lagoons, bubble columns, pilot tanks, earthen pots, basins, and concrete tanks (Figure 1) [13,20,190]. The cultivation system should have a short path length, adequate microalgae culture for proper mixing, and full light dispersion [191]. Microalgae growth and product formation do not occur simultaneously. To overcome this challenge, microalgae are grown under a two-stage cultivation system, where microalgae are grown initially under nutrient-rich conditions to achieve maximum biomass productivity, followed by nutrient-deprived conditions for cellular morphogenesis and stimulation of value-added products [192,193]. Proteins, carbohydrates, and lipids are synthesized during the growth phase, while amelioration of these products occurs under nutrient-deprived and stress conditions [8]. Light [194], temperature [195], pH [196], salinity [197], trace metals [198], Ultraviolet Radiation [199], and dark–light photoperiods are the important key factors essential for enhanced biomass production and targeted biomolecules [52]. *Chlorella vulgaris* grown under nitrogen-rich conditions achieved a maximum lipid productivity of 71.1 mg L^−1^ d^−1^ [200]. *Chlorococcum*, a cosmopolitan microalga tolerant to extreme pH and temperature, produces astaxanthin, canthaxanthin, lutein, β-carotene, adonixanthin, and cis-isomers of ketocarotenoid [201]. *H. Plulvalis* accumulates 95% of total carotenoids and increased fatty acid biosynthesis due to improvements in environmental conditions (light strength, temperature, salinity, and nutrient limitation) [202]. Although reduced amounts of nutrient have shown propitious results, utilizing wastewater as a nutrient source has been considered promising and an alternative to economic cultivation [203,204]. Nitrates and phosphates can serve as essential nutrients while acetate can be utilized as organic carbon for algal cultivation [205,206]. Apart from external stress conditions, researchers have now targeted genetic engineering and manipulation of metabolic pathways that redirect cellular functions in the synthesis of compounds of interest, enlarging the competences of microalgae. Homologous recombination, RNA silencing, selection marker, biolistic transformation, artificial hybrids, somatic variants, and mutant’s development are communal tools used in genetic engineering for pharmaceutical and nutraceutical products of monetary value [5,20]. To date, over 40 different microalgae species have been successful in genetic manipulation including the green algae *Chlorellavulgaris*, *Haematococcus pluvialis*, *Dunaliellasalina*, *Chlamydomonas reinhardtii*, and the Bacillariophyceae *Phaeodactlyum tricornutum and Thalassiosira pseudonana* [207]. Today, microalgae represent a viable source for recovery of high value-added products and have drawn attention for their use in health products in addition to biofuels.

## 7. Harvesting and Downstream Process of Microalgae

The downstream method entails lower-cost exploitation of high-value microalgae metabolites with high product recovery. The composition and productivity of microalgae mainly depends on cultivation mode, nutrient composition, and harvesting time [208]. The most commonly used harvesting techniques for performance, operating economy, and technological feasibility are membrane filtration, centrifugation, microfiltration, ultrafiltration, gravity sedimentation, electrolytic phase, electrophoresis, and magnetic separation [209]. Harvesting of microalgae is challenging as these are microscopic (3–30 μm), with low sedimentation rates and negative surface charges, which cause repulsion between the cells. Gravitational sedimentation and filtration are widely employed to harvest relatively large (>70 μm) microalgae *Spirulina* and *Coelastrum* [210]. However, microalgae with dimensions (<30 μm), such as *Scenedesmus*, *Dunaliella*, and *Chlorella*, often blind the filter, and frequent replacement results in high operational costs [211]. The preferable harvesting method is chosen according to the attributes of the microalgae (size, species, and cell density) and the final product. Conjoining chemical or biological coagulation/flocculation harvesting methods will improve process productivity and minimize operational costs [212]. The microalgae cell wall is a complex and tri-layered structure typically composed of polysaccharides (cellulose, pectin, and xylan), proteins (glycoproteins), and sugars, making them highly recalcitrant to degradation [213]. The cell wall has high mechanical strength and chemical resistance due to the presence of covalent, hydrogen, and van der Waals forces [214]. The exposure of microalgae cells to harsh shear levels, high temperatures, and pressure may change the integrity, loss of functionality, and subsequent release of the compound. For example, microalgal cell wall tensile strength is up to 9.5 MPa, which is three times that of *Daucus carota* [215]. It is important to use mild pre-treatment procedures to promote the recovery of the delicate intracellular compounds from the microscopic cell. Microalgae cells can be pre-treated/disrupted individually or in amalgamations, i.e., (1) mechanical/physical, (2) chemical, and (3) enzymatic/biological pre-treatment method (Figure 1).

### 7.1. Mechanical/Physical Pre-Treatment 

Mechanical/physical cell disruption involves the breakdown of the cell wall to expedite the entry of solvents and solubilize intracellular complexes. Pre-treatment of microalgae cells can be carried out using bead milling, steam explosion, ultrasonication, and microwave-assisted extraction. Steam explosion is a cost-effective and efficient cell disruption method. Lorente et al., reported that *N. gaditana*, *C. sorokiniana*, and *P. tricornutum* achieved the highest lipid extraction yields using steam explosion [216]. Glass beads, zirconia-silica, zirconium oxide, or titanium carbide with 0.5 mm diameter is effective in microalgae disruption for the release of intracellular compounds. Bead milling of *C. vulgaris* releases water-soluble proteins with yields ranging between 32% to 42% and energy consumption below 2.5 kWhkg^−1^ dry weight [217]. Bead milling perturbation of *H. pluvialis* improves the recovery of astaxanthin by up to 98% during extraction at 400 rpm for 5 min [218]. Ultrasound-assisted extraction (UAE) is an efficient, economical, and rapid extraction method for the disruption of cell walls [219]. It works with low amounts of solvents and low temperatures to preserve thermolabile compounds and structural properties of the by-product. Polyphenols, antioxidants, and phycobiliproteins are the main compounds extracted from microalgae using UAE. In the destruction of cell membranes and the extraction of separate intracellular compounds, the energy strength of 0.4 kWh L^−1^ gained worldwide interest [220]. The UAE process obtained a yield of 15.6% phycocyanin in 20 min in *Spirulina platensis*, while the extraction of Soxhlet took 4 h to extract 11.1% [221]. Microwave-assisted extraction (MAE) yields high-value biological compounds by dissipating electromagnetic waves in the irradiated medium [222]. MAE has been used for the pretreatment of a variety of microalgae classes including *Chlorophyceae*, *Bacillariophyceae*, *Eustigmatophyceae*, *and Phaeophyceae* [223]. MAE is widely employed to extract bioactive compounds such as alkaline galactans, carrageenans, polysaccharide, and agar from *Chlorella*, *Haematococcus*, Bacillariophyceae, and seaweeds with high mechanical resistance and silica frustules [224,225].

### 7.2. Chemical Pre-Treatment

Chemical pre-treatments are easy with less energy inputs and high scalability but the major drawback is chemical toxicity and product recovery. Chemical solvents, salts, bases, ozone, and ionic liquids are widely used to extract bioactive compounds from algal biomass [226]. For their use in pharmaceuticals/nutraceuticals, the US Food and Drug Administration (FDA) categorized solvents into different groups and proposed allowed daily exposure (PDE) limits for each solvent. Chloroform PDE, for example, is equivalent to 0.6 mgday^−1^, whereas hexane can be used up to 2.9 mgday^−1^ [227]. Chemical treatment of *Thraustochytrids* biomass for astaxanthin extraction showed higher yields with inorganic acids compared to organic ones [228]. Ionic liquids are liquid-state molten organic salts composed of an asymmetric cation and inorganic anion. Owing to their synthetic stability, solubility, conductivity, polarity, and hydrophobicity in the range of cationic or anionic constituents, these are known as designer solvents [22]. Ionic liquids break down complex networks of lignin, cellulose, and hemicellulose due to their high capacity to accept hydrogen bonds [229]. Imidazolium- and cholinium-based ILs are widely investigated for their potential to extract bioactive compounds from *Synechocystis* sp., *Scenedesmus* sp., *C. vulgaris*, *and Spirulina* [230].

Supercritical fluid extraction (SFE) is a promising technology for green extraction, focused on the use of solvents above their critical temperatures and pressures. SFE is popularly used for the recovery of lipids from microalgae [226]. Different solvents such as ethanol, methanol, hexane, butane, and sulfur hexafluoride are used for the abstraction of biological composites using CO_2_ as a supercritical fluid. SCF-CO_2_ easily infiltrates the microalgae cell wall due to its high permeability and diffusivity that solubilizes intracellular compounds [231]. Sanzo et al., stated that astaxanthin and lutein yield was enhanced to 98.6% and 52% by varying the pressure from 10 to 50 MPa at 50 °C and CO_2_ flow rate of 14.48 gmin^−1^ in *H. pluvialis* [35]. Increased extraction time, temperature, and pressure increased the recovery of 79.6% DHA from *Nannochloropsis* sp. [232]. A similar phenomenon was observed by Molino et al., with the isolation of carotenoids from *D. salina* [233]. Pressurized Fluid Extraction (PFE) is a technique that uses high temperature (below critical point) and pressure during the extraction process to retain the solvent in a liquid state [234]. Water is used as extraction solvent and an alternative to organic solvents in Pressurized Liquid Extraction (PLE) because it has the same similar dielectric constant (ethanol or methanol) and solvent characteristics to that of organic solvents. Carotenoids are widely extracted compounds through PLE method. Ethanol/acetone/water has shown promising results as solvents in the PLE method for the extraction of different kinds of carotenoids from *Phormidium* sp., *Phaeodactylum tricornutum*, *Chlorococcum* sp., *Neochloris oleoabundans*, *Scenedesmus* sp., and *C. vulgaris* [235,236].

### 7.3. Enzymatic/Biological Pre-Treatment

Enzymatic disruption is a non-destructive pre-treatment method to facilitate the access of microalgae cellular components. It is a cost-intensive process, is highly effective, and operates under mild environmental conditions with less energy demand [237]. During lysis, enzymes act selectively, lyse, and degrade specific chemical linkage without destroying particle existence or any non-specific reaction in the solution. For cell lysis, lytic enzymes such as glycosidases, glucanases, cellulose, pectinase, and lipases have been used extensively [214]. Tang et al., reported that the utilization of carbohydrases disrupts the cell wall and enhances protein yield from rice bran [238]. Papain hydrolyzed from *N. incerta* demonstrated higher antioxidant activity relative to alcalase, neutrase, alpha-chymotrypsin, pepsin, pronase-E, and trypsin [239]. Recent studies demonstrated that the simultaneous use of enzymes facilitates the extraction of intracellular compounds. For example, cellulase hydrolyzes the cellulosic structure while lysozyme degrades resilient polymers present on the microalgae envelope [240]. The combination of chemical or mechanical treatment with enzymatic cell disruption may minimize the expense of the enzymatic procedure, the use of solvents, energy consumption, and improve the efficiency of intracellular material recovery. Emerging cell destruction developments that are still under development are explosive decompression, microfluidization, pulsed arc technology, and disruption using cationic polymer-coated membranes.

## 8. Conclusions and Future Perspective

Bioactive metabolites of microalgal origin have gained the biotechnological spotlight to decelerate the rate of malnourishment in developing nations. Even today, 1 out of 10 is undernourished due to malnutrition. Bioactive compounds obtained from microalgae, such as polyunsaturated fatty acids, carotenoids, phycobiliproteins, polysaccharides, antioxidants, sterols, and other secondary metabolites, have significant potential through a sustainable approach to meet increasing global demands. Microalgae metabolites act as the repository of natural antibiotics benefitting animal/aquaculture and human health by replacing antimicrobial compounds of synthetic origin. Microalgae are more nutritious, productive, and could be the next alternative for traditional sources of animal and aquatic feed with enriched eggs, meat, and milk. Microalgae-derived bioactive metabolites are renowned for antioxidant and broad-spectrum anti-inflammatory and antibacterial properties with impressive safety profiles, which help in the reduction and prevention of diseases associated with oxidative stress. The application of bioactive compounds proved to be beneficial and much more effective when compared to present traditional treatments and therapies. These metabolites act widely against emerging infectious diseases, antibiotic resistant bacteria, viral infections (epidemic and pandemic), and can prevent the delay or onset of chronic and carcinogenic diseases. Although numbers of products isolated from microalgae are commercially available for human health and nutrition, the social acceptance and awareness of health benefits of microalgae are still lacking. When eaten entirely, the chemical constituents of microalgae can exert synergistic results. In order to exploit the pharmacological and biological properties of algal-derived materials, numerous in vivo clinical studies are also required. Microalgae cultivation in different wastewater streams using genetically modified techniques explores their biotechnological potential to favor bio-based process. Sequential or co-extraction of multiple bioactive compounds with low energy consumption and high yields needs to be prioritized. Nonetheless, more studies are needed to turn the current technology into green technology for the exploitation of microalgae in bioengineering and fulfill the requirements compared to plants.

## Figures and Tables

**Table 1:** Microalgae bioactive metabolites and producers for human nutrition.

S. No.	Microalgae Compounds	Pigment	Microalgae Species	Activity on Human Health	Reference
1.	Chlorophyll	Chlorophyll	*Chlorella* sp., *Sanropus androgynous*, Green algae	Food additive, antioxidant activity, immune activators, cytotoxic towards tumoral cells,	Khanra et al., 2018 [28]; Koller et al., 2014 [64]; Mishra et al., 2011 [27]; Odjadjare et al., 2017 [26]; www.oligae.com (accessed on 15 March 2021)
2.	Carotenoids	β-carotene	*Botryococcus braunii*, *Chlamydomonas nivalis*, *Chlamydocapsa* sp., *Chlorella sorokiniana*, *Chlorococcum* sp., *Chondria striolata*, *Dunaliella salina*, *Dunaliella tertiolecta*, and *Paeonia obovate*	Anti-aging, cancer, immune control, coronary disease prevention, retinal and sensory disability enhancement and low-density lipoprotein oxidation inhibition	Andrade et al., 2018 [56]; Barkia et al., 2019 [12]; Galasso et al., 2019 [30]; Gong and Bassi 2016 [31]
		Astaxanthin	*Ankistrodesmus braunii*, *Chlamydomonasnivalis*, *Chlorella vulgaris*, *Chondria striolata*, *Haematococcus lacustris*, *Haematococcus pluvialis*, *Haematococcus* sp., *Monoraphidium* sp., *Scenedesmus obliquus*	Cancer defense, inflammation, metabolic syndrome, diabetes, neurodegenerative and ocular diseases, lung injury, repressed alveolar wall swelling and myeloperoxidase activity	Cai et al., 2019 [65]; Capelli et al., 2019 [32]; Chang et al., 2010 [66]; Galasso et al., 2019 [30]; Park et al., 2010 [67]; Talukdar et al., 2020 [33]; Wu et al., 2014 [68]
		Lutein	*Ankistrodesmus braunii*, *Chlamydomonas acidophila*, *Chlorella fusca*, *Chlorella sorokiniana*, *Chlorococcum* sp., *Tetraselmis suecica*	Antioxidant and anticancer activity, prevents macular degeneration, Cataract, atherosclerosis, diabetic retinopathy, and age-related retinal degeneration	Liu et al., 2017 [69]; Rasmussen and Johnson 2013 [70]; Sansone et al., 2017 [71]
		Violaxanthin		Anti-proliferative activity	Koller et al., 2014 [64]
		Canthaxanthin	*Chlamydocapsa* sp., *Chlamydomonas* *nivalis*, *Chlorella vulgaris*, *Chlorococcum* sp., *Chlorella zofingiensis*, *Neospongiococcum* sp.	Increases Vitamin E, antioxidative, anti-inflammatory and neuroprotective properties	Sathasivam et al., 2019 [41]
		Fucoxanthin and Zeaxanthin	*Chaetoceros gracilis*, *Chlamydomonasnivalis*, *Dunaliella salina Isochrysis* sp., *Ochromonas* sp., *Odontella aurita*, *Phaeodactylum tricornutum*, *Prymnesium parvum*, *Salpingoeca marina*	Anti-cancer and anti-proliferative properties, prevention of osteoporosis, rheumatoid arthritis and diabetic diseases, suppressing insulin and hyperglycemia	Gong et al., 2016 [31]; Liu et al., 2017 [69]
3.	Polysaccharides	Polysaccharides	*Chlorella stigmatophora*, *Phaeodactylum tricornutum*, *Porphyridium cruentum*, *Rhodella reticulata*	Antioxidant and tumoricidal activity, Reduces free radicals, atherosclerosis, blood cholesterol, gastric ulcers, sores, constipation and hypercholesterolemia	Chen et al., 2018 [45]; de Gardeva et al., 2009 [47]; Morais et al., 2015 [46]
		Sulfonated polysaccharides	*Chlorophyta*, *Phaeophyta* and *Rhodophyta*	Anticancer, antifungal, hepatoprotective, antihelminthic, anti-protozoal, anti-inflammatory, anti-coagulant, immunomodulation and enhanced skin tissue regeneration. Reducing coronary heart disease and acts as coating material on the sanitary items for COVID-19 prevention	Gaikwad et al., 2020 [49]; Lekshmi and Krupa 2019 [48]; Morokutti-Kurz et al., 2017 [72]; Olasehinde et al., 2017 [73]; Raposo et al., 2014 [74]
4.	Polyunsaturated Fatty Acids (PUFAs)		*Chaetoceros calcitrans*, *Crythecodinium cohnii*, *Isochrysis galbana*, *Monodus subterraneus*, *Nannochloropsis* sp., *Pavlova salina*, *Phaeodactylum tricornutum*, and *Porphyridium cruentum*	Reduces occurrence of chronic diseases such as obesity, arthritis, diabetes, cardiovascular diseases, hypercholesterolemia and improves brain function	Bhalamurugan et al., 2018 [42]; Katiyar and Arora 2020 [40]; Mourelle et al., 2017 [37]
		Eicosapentaenoic acid (EPA)	*Chlorella vulgaris*, *Nannochloropsis* sp., *Pavlova* sp., *Tetraselmis* sp.	Immune activator, blood clotting, regulation of blood pressure and prevents thrombosis, atherosclerosis and beneficial for coronal heart diseases	Charles et al., 2019 [43]; Levassuer et al., 2020 [11]
		Docosahexaeonic acid (DHA)	*Crypthecodiuimu* sp., *Pyramimonas* sp., *Schizochytrium* sp., *Thraustochytrid strain 12B*	Anti-inflammatory, anticancer function, used in food for pregnant, nursing and cardiovascular patients as dietary supplements	Long et al., 2018 [75]; Raghukumar, 2008 [76]; Teng et al., 2015 [77]
		Arachidonic acid (ARA)	*Navicula atomus*, *Pediastrum boryanum*, *Porphyridium* sp.	Platelet aggregators, vasoconstrictor, vasodilators and have antiaggregative action on the endothelium in neutrophils	de Morais et al., 2015 [46]; Paliwal et al., 2017 [52];
		γ-linoleic acid (GLA)	*Anthrospira*, *Chlorococcum* sp., *Dunaliella primolecta*, *Spirulina* sp.	Relieves from breast cancer, skin allergies, alcoholism, obesity, rheumatoid arthritis, blood pressure, heart diseases, premenstrual syndrome, sclerosis, hyperactivity attention deficit disorder (ADHD), diabetes-related neural issues	Koller et al., 2014 [64]; Mourelle et al., 2017 [78]
		Linolenic acid	*Botryococcus braunii*, *Chlorococcum* sp., *Dunaliella primolecta*, *Scenedesmus obliqus*, *Tetraselmis suecica*	Anti-inflammatory, acne reductive and moisture retention	Day et al., 2009 [79]
5.	Proteins	Glycoprotein	*Alexandrium minutum*, *Chlorella* sp., *Dunaliella* sp., *Schizochytrium* sp., *Spirulina*	Antihypertensive and Angiotensin I inhibitory activities, appetite suppression and reduction of LDL-cholesterol. It is also used as dietary supplements (tablets, powder and paste)	Andrade et al., 2018 [56]; Caporgno and Mathys 2018 [50]; da Silva Viz et al., 2016 [17]; Galasso et al., 2019 [30]; Hempel et al., 2011 [51]; Kim et al., 2012 [80];Marquez-Escobar et al., 2018 [81]
		Phycobiliprotein	Cyanobacteria	Bio-sensor, neuroprotective, anti-nephrolithe, anti-hyperglycemic, immunomodulatory and hepatoprotective properties. Used as fluorescent labels in antibodies and receptors, flow-cytometry, immunohistochemistry	Bhattacharjee 2016 [82]; Paliwal et al., 2017 [52]
		Recombinant protein	*Chlamydomonas elipsoidea*, *Chlamydomonas reinhardtii*;	Anemia treatment, wound healing, anti-malarial vaccines, antibody against anthrax, Herpes simplex virus, human papilloma virus, white spot syndrome virus, foot and mouth disease virus, immunotoxins against B-cell lymphoma and increase resistance to UV-induced stress	Brasil et al., 2017 [10]; Hempel et al., 2016 [53]; Specht et al., 2010 [55]; http://www.olson.com.br/ (accessed on 15 March 2021)
6.	Amino acid	Mycosporine-like amino acid (MAA)	*Chlorella pyrenoidosa*, *Chlorella vulgaris*, *Microcystis aeruginosa*, *Nitzschia incerta*	Antioxidant properties, prevents atherosclerosis, cancer, coronary diseases and used in photo-aging protective formulations	Gregory et al., 2013 [54]; Kim et al., 2011 [83]; Lawrence et al., 2018 [84]
7.	Vitamins	Vitamin A	*Chlorella* sp., *Eisenia arborea*, *P. cruentum*	Involved in vision, reproduction, immune function and cellular communication	Andrade et al., 2018 [56]; Koyande et al., 2019 [14];
		Vitamin B	*Chlorella*, *Spirulina*, *Pavlova*, *Tetraselmis*	Anticancer activity, reduces cholesterol, regeneration of blood cells, DNA repair, histone methylation, preservation of skin and mucous membranes and cardiovascular disease	Becker et al., 2004 [85]; Delasoie et al., 2018 [86]
		Vitamin E	*Chaetoceros calcitrans*, *Dunaliella tertiolecta*, *Nannochloropsis oculata*, *Porphyridium cruentum* and *Tetraselmis suecica*	Protects membrane lipids from oxidative damage and prevent coronary, atherosclerosis as well as neurodegenerative diseases. Improves endothelial function, vascular health and inhibits prostate cancer cell growth	Bong et al., 2013 [60]; Giammanco et al., 2015 [59]
		Vitamin K	*Anabaena cylindrica*	Protect against toxic pollutants, prevention of chronic diseases	Tarento et al., 2018 [58]
8.	Sterols	Brassicasterol Stigmasterol Phytosterols	*Chlorella vulgaris*, *Pavlova lutheria*, *Nanochloropsis* sp. *BR2* and *Tetraselmis* sp. *M8*	Anti-inflammatory and anticancer activities, stabilizes phospholipid bilayers, reducing blood cholesterol levels in hyper and normocholesterolemic people and inhibit colon cancer development	Ahmed et al., 2015 [63]; Lopes et al., 2013 [61]; Luo et al., 2015 [62]

**Table 2:** Biological extracts and therapeutic applications of different microalgal species against human pathogens.

Antibacterial Activity				
**S. No.**	**Microalgae species**	**Compound/Fraction**	**Targeted Microorganism**	**References**
1.	*Bacillariophyceae* and *Chrysophyceae*	Methanolic extracts, lysed cells, phycobiliproteins, beta-ionone and neophytadiene	*Escherichia coli*, *Pseudomonas aeruginosa*, *Salmonella typhimurium*, *Enterobacter aerogenes*, *Klebsiella pneumoniae*, *Vibrio cholera* and *Proteus vulgaris*	Cannell et al., 1988 [100]; Falaise et al., 2016 [101]; Mudimu et al., 2014 [102]; Najdenski et al., 2013 [97]
2.	*Chlorella* sp.	Chlorellin	Gram-positive (G+) and Gram-negative (G−) bacteria	Pratt et al., 1944 [96]
3.	*Fischerella ambigua*	Parsiguine	*S. epidermidis* PTCC 1114 and *C. krusei* ATCC 44507	Najdenski et al., 2013 [97]
4.	*Chlorococcum* strain HS-101, *Dunaliella primolecta*, *Phaeodactylum tricornutum*	Eicosapentaenoic acid (EPA), hexadecatrienoic acid (HTA) and palmitoleic acid (PA)	Methicillin-resistant *S. aureus* (MRSA), *B. subtilis*, *Bacillus cereus*, *S. aureus*, *Enterobacter aerogenes*	Desbois et al., 2008 [103]
5.	*Euglena viridis*	Organic solvent extracts	*Edwardsiella tarda*, *Aeromonas hydrophila*, *Pseudomonas tarda* *Pseudomonas fluorescens*, *Pseudomonas aeruginosa*, *Vibrio alginolyticus putida*, *V. anguillarum*, *Vibrio alginolyticus*, *Vibrio anguillarum* *Vibrio harveyi*, *fluvialis*, *Vibrio parahaemolyticus*	Das et al., 2005 [161]
6.	*Scenedesmus obliquus*, *Haematococcus pluvialis*	Fatty acids	*E. coli* and *S. aureus*	Rodríguez-Meizoso et al., 2010 [104]
7.	Marine microalgae	Decadienal	Human pathogens MRSA and *Haemophilus influenza*	Mostafa et al., 2012 [89]; Smith et al., 2010 [98]
8.	*Spirogyra grantiana* and *Oscillatoria sancta*	Ethanolic and methanolic extract	*E. coli*, *P. vulgaris* and *P. mirabilis*	Prakash et al., 2011 [105]
9.	*Dunaliella salina* and *Pseudokirchneriella subcapitata*	Methanolic extract	*S. aureus*, *P. aeruginosa*, *Escherichia coli* and *Klebsiella* sp.	Pane et al., 2015 [106]
10.	*Porphyridium aerugineum*	Phycobiliproteins	*S. aureus*, *B. subtilis*, *S. pyogenes*	Shannon et al., 2016 [95]
**Antifungal Activity**				
**S. No.**	**Microalgae species**	**Compound/Fraction**	**Targeted microorganism**	**References**
1.	*Chaetoceros* sp., *Chlorella vulgaris*, *Haematococcus pluvialis*, *Porphyridium purpureum*, *Rhodella reticulata*, *Scenedesmus quadricauda*	Polysaccharides, organic solvent extracts, pigments and lipid fractions	*A. fumigatus*, *A. niger*, *Penicillium* sp., *C. albicans*, *C. neoformans*, *S. cerevisiae*, *Microsporum* sp., *E. floccosum* and *T. mentagrophytes*	Gueho et al., 1977 [123]; Mudimu et al., 2014 [102]; Washida et al., 2006 [122];
2.	*Gambierdiscus toxicus*, *Thalassiothrix frauenfeldii*	Gambieric acid	*Penicillium*, *Aspergillus oryzae*, *Penicillium chrysogenum citrinum*, *Variotii paecilomyces*, *T. Mentagrophytes*	Walter and Mahesh 2000 [124]
3.	*Chlorococcum humicola*, *Porphyridium aerugineum*	Beta-carotene, chlorophyll-a and b	*C. albicans*, *A. flavus* and *A. niger*	Bhagavathy et al., 2011 [162]
4.	*Amphidinium klebsii*	Polyols: karatungiols A(1)	*A. niger*	Ghannoum et al., 1999 [125]
5.	*Haslea. karadagensis*	Pigments	*Corollospora maritima*, *Dendryphiella salina* and *Lulworthia* sp.	Gastineau et al., 2012 [126]
6.	*Chaetoceros lauderi*, *Chlorella vulgaris* and *Gambierdiscus toxicus*	Organic extracts	*Aspergillus fumigatus*	Ghasemi et al., 2007 [127]
7.	*Scenedesmus quadricauda*	Organic solvent extracts	*C. albicans*, *S. cerevisiae*, *A. flavus*, *A. niger*, *P. herquei*, *A. brassicae*, *F. moniliforme*, *Helminthosporium* sp.	Alangaden 2011 [119]
**Antiviral Activity**				
**S. No.**	**Microalgae species**	**Compound/Fraction**	**Targeted virus**	**References**
1.	*Gelidium cartilagenium*	Polysaccharides	Influenza B and mumps viruses	Gerber et al., 1950
2.	*Dunaliella* sp., *Spirulina platensis*	Acyclovir^®^, Spirulan	Type 1 Herpes simplex virus (HSV-1) and Type 1 Human Immunodeficiency Virus (HIV-1)	Hayashi et al., 1996 [111]; Raposo et al., 2014 [74]
3.	*Arthrospira platensis*, *Cochlodinium polykrikoides*, *Gymnodinium impudicum Porphyridium* sp., and *Rhodomonas reticulate*	Polysaccharides	*Varicella zoster viruses* (VZV), human cytomegalovirus (HCMV), murine leukemia virus (MuLV), Flu-A viruses, Hepatitis B virus (HBV); viral hemorrhagic septicemia virus (VHSV); African swine fever virus (ASFV), vaccinia virus (VACV), vesicular stomatitis virus (VSV), Encephalomyocarditis virus, Flu-A and Flu-B, respiratory syncytial virus types A (RSV-A) and B (RSV-B), measles, mumps	Falaise et al., 2016 [101]; Huleihel et al., 2002 [114]; Raposo et al., 2014 [74]; Santoyo et al., 2011 [113]; Silva et al., 2018 [112]; Talyshinsky et al., 2002 [115]
4.	*Gyrodinium impudicum*	Sulphated polysaccharide	Encephalomyocarditis RNA virus (EMCV)	Huheihel et al., 2002 [114]; Kim et al., 2012 [80]
5.	*Arthrospira platensis*, *Porphyridium. purpureum*	Polysaccharide TK-V3 Exopolysaccharides	Vaccinia and Ectromelia orthopoxvirus	Radonic et al., 2010 [116]
6.	*Cochlodinium polykrikoides Navicula directa*	Naviculan	HIV-1,HSV-1 and influenza virus type A (IFV-A)	Lee et al., 2006 [117]
7.	*Chlorella autotrophica*, *Dunaliella tertiolecta*, *Ellipsoidon* sp., *Isochrysis galbana var. Tiso* and *Porphyridium cruentum*	Endocellular extracts	Inhibited the viral infection of epithelioma papulosum cyprinid (EPC) cells	Graf et al., 2018 [118]; Talyshinsky et al., 2002 [115]
8.	*Dunaliella salina*, *Haematococcus pluvialis*	Short chain fatty acids, β-ionone, phytol, neophytadiene, palmitic and α-linolenic acids	Herpes simplex virus type 1 (HSV-1)	Santoyo et al., 2011 [113]
9.	*Cochlodiniumpolykrikoides*	Extracellular sulphated polysaccharides: A1 and A2	Flu-A and B-Flu, Syncytial breathing Forms of Viruses A (RSV-A) A, and B (RSV-B), HIV-1, HSV-1, Parainfluenza type 2(PFluV-2)	Hasui et al., 1995 [163]; Morokutti-Kurz et al., 2017 [72];
**Antioxidant Activity**				
**S. No.**	**Microalgae species**	**Compound/Fraction**	**Application**	**References**
1.	*Anabaena flos aquae*, *Anabaena oryzae*, *Nostoc humifusum*, *Chlorella vulgaris*, *Nostoc muscorum*, *Oscillatoria* sp., *Phormedium fragile*, *Spirulina platensis*, and *Wollea saccata*	Algal extracts	Antioxidant activity is higher compared to standard Butylated hydroxytoluene (BHT) antioxidant	Barkia et al., 2019 [12]; Kattappagari et al., 2015 [87]; Lawrence et al., 2018 [84]; Shanab et al., 2012 [90]
2.	*Chlorella sorokiniana* *Sanropus androgynous*	β-carotene, ά tocopherol, Chlorophyll and lutein	High radical scavenging activity	de Morais et al., 2015 [46]; Matsukawa et al., 2000 [92]; Suparmi et al., 2016 [91];
3.	*Porphyridium cruentum*	Exopolysaccharides	Scavenging hydroxyl, superoxide anion, and DPPH free radicals	Li et al., 2007 [164]; Wu et al., 2014 [68]
4.	*Dunaliella* sp., *Rhodella reticulata*	Glutathione, Sulfated polysaccharides	Scavenges ROS and prevents cellular damage	de Jesus Raposo et al., 2013 [94]; Li et al., 2007 [164]; Mostafa 2012 [89]; Raposo et al., 2014 [74]
**Anticancer Activity**				
**S. No.**	**Microalgae species**	**Compound/Fraction**	**Application**	**References**
1.	*Nostoc muscorum*	Phycobilins, phenolic compounds and polysaccharides	Anticancer activity against cell lines of Ehrlich Ascites Carcinoma and Human hepatocellular cancer	Shanab et al., 2012 [90]; Sheih et al., 2010 [136]
2.	Red algae	c-phycocyanin	Anti-proliferation	Kim et al., 2011 [83]
3.	*G. impudicum*	sulphate polysaccharide (p-KG03)	Prevents tumor cell growth	Wang et al., 2007 [132]
4.	*Chlorella vulgaris*	β-(1,3)-glucan	Antitumor agent	Delasoie et al., 2018 [86]; Laroche and Michaud 2007 [133]
5.	*Porphyridium* sp.	Sulphated polysaccharides	Tumor cell inhibition and proliferation of colon cancer in rats	Geresh et al. 2002 [135]; Sun et al. 2012 [134]
